# Prognostic features and comprehensive genomic analysis of *NF1* mutations in *EGFR* mutant lung cancer patients

**DOI:** 10.1002/cam4.4925

**Published:** 2022-06-14

**Authors:** Hong‐xia Tian, Zhi‐hong Chen, Guang‐Ling Jie, Zhen Wang, Hong‐hong Yan, Si‐pei Wu, Shui‐lian Zhang, Dan‐xia Lu, Xu‐chao Zhang, Yi‐long Wu

**Affiliations:** ^1^ Guangdong Lung Cancer Institute, Guangdong Provincial People's Hospital Guangdong Academy of Medical Sciences Guangzhou China; ^2^ Guangdong Provincial Key Laboratory of Translational Medicine in Lung Cancer, Guangdong Provincial People's Hospital Guangdong Academy of Medical Sciences Guangzhou China

**Keywords:** epidermal growth factor receptor, lung cancer, molecular typing, next‐generation sequencing, *NF1* gene, overall survival, *TP53* gene

## Abstract

**Objective:**

*NF1* is a tumor suppressor gene that encodes the neurofibromin protein and negatively regulates Ras signaling. This study was aimed to investigate the molecular, clinical characteristics, and prognostic features of *NF1* gene in *EGFR* mutant lung cancer patients.

**Method:**

The next‐generation sequencing (NGS) was used to analyze the data from lung cancer patients in the Guangdong Lung Cancer Institute (GLCI) from June 2016 to December 2020.

**Results:**

Somatic *NF1* mutations were present in 4.2% (135/3220) of Chinese lung cancer patients. *NF1* mutations where clearly enriched in older (*p* < 0.001), male (*p* < 0.001), and smoking (*p* < 0.001) patients. Patients with *NF1* mutations were more likely to have *TP53* (*p* = 0.003), *BRAF* (*p =* 0.001) and *RASA1* (*p* = 0.026) mutations and mutually exclusive with *EGFR* mutations (*p* = 0.006). *TP53* mutation had worsen prognosis in cases of *NF1* mutant (*p* = 0.026) or *EGFR*/*NF1* co‐mutant (*p* = 0.031) lung adenocarcinomas (LUAD) patients. There was no effect on overall survival (OS) in LUAD patients with and without *NF1* mutations, even in LUAD driver‐gene negative patients. *NF1*/*EGFR* co‐mutation patients had a longer OS than a single mutation of either the *EGFR* gene (median OS: 47.7 m vs. 30.2 m, hazard ratio [95% CI], 0.47 [0.30–0.74], *p* = 0.004) or *NF1* gene (47.7 m vs. 19.0 m, 0.44 [0.27–0.73], *p* = 0.003). Furthermore, *NF1* mutations significantly prolonged OS in *EGFR* mutant/*TP53* wild‐type LUAD patients (106.5 m vs. 25.5 m, 0.28 [0.13–0.59], *p* = 0.003) but not in patients with *EGFR*/*TP53* co‐mutations (36.8 m vs. 30.2 m, 0.70 [0.39–1.26], *p* = 0.280).

**Conclusion:**

Our results indicated *NF1* mutations served as a good prognostic factor in *EGFR* mutant/*TP53* wild‐type lung cancer patients in this single‐center study. *TP53* mutation was obviously enriched in *NF1* mutant patients and had shorter OS.

## Graphical abstract

This was the largest sample size analysis for *NF1* gene in East Asia lung cancer patients. *NF1* mutant tumors could define a specific population with a distinct clinical and molecular profiles. Our results showed for the first time that *NF1* mutations significantly prolonged overall survival in *EGFR* mutant/*TP53* wild‐type lung adenocarcinoma (LUAD) patients and served as a good prognostic factor. *TP53* mutations were clearly enriched in the *NF1* mutant lung cancer patients, and had worsen prognosis in cases of *NF1* mutant or *EGFR*/*NF1* co‐mutant LUAD patients.

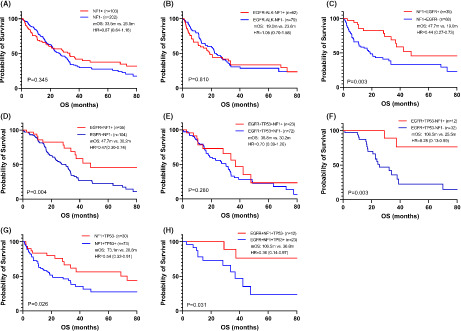

**What's new?**

The molecular, clinical characteristics and prognostic features of *NF1* gene in *EGFR* mutant lung cancer patients have not been extensively explored. We analyzed *NF1* mutations in a large‐scale cohort of Chinese lung cancer patients. We reported that The frequency of *NF1* mutations was significantly lower in the Chinese population than western populations. *NF1* mutant tumors could define a specific population with distinct clinical and molecular profile. *NF1* mutations were no effect on overall survival in lung cancer patients, but maybe a potential biomarker for good prognosis to *EGFR* mutant/*TP53* wild‐type lung adenocarcinoma patients. *TP53* mutation was obviously enriched in cases of *NF1* mutant patients and had shorter overall survival. The study would provide more information for exploring biomarker of lung cancer.

## 1 INTRODUCTION

Molecular targeted therapies have dramatically improved treatment for patients whose tumors harbor somatically activated oncogenes, such as epidermal growth factor receptor (*EGFR*) mutations, anaplastic lymphoma kinase (*ALK*) rearrangements, and other mutations.^1^, ^2^ Additional molecular targets and drug resistance mechanisms using targeted therapy in lung cancer must be investigated.

The *NF1* gene is a tumor pathogenic suppressor gene located on chromosome 17q11.2. Mutation inactivation was initially found in patients with the common inherited tumor predisposition syndrome neurofibromatosis type I (NF1).^3^, ^4^ The *NF1* gene is one of the largest genes in the human genome; it encodes a Ras GTPase‐activating protein (neurofibromin) composed of 2839 amino acids.^5^, ^6^ The neurofibromin protein inhibits tumor growth by negatively regulating the RAS proto‐oncogene. The inactivation of *NF1* function has an important role in carcinogenesis that involves the hyperactivation of wild‐type RAS proteins and dysregulation of the RAS/MAPK pathway, in which GTP‐bound RAS activates the RAF–MEK–ERK signaling cascade to control proliferation.^7^ Activated RAS‐GTP also stimulates PI3K/AKT signaling, which protects cells from apoptosis. Acquired somatic *NF1* mutations have been identified in various sporadic malignancies that were not associated with NF1,^8^ including lung cancer,^9^ ovarian cancer,^10^ breast cancer,^11^ and acute myeloid leukemia.^12^ The Cancer Genome Atlas (TCGA) Research Network reported that *NF1* gene mutations are related to the development of lung cancer. Whole‐exon sequencing showed that the mutation rate of the *NF1* gene in lung cancer was 11%; it was enriched in samples otherwise lacking oncogene mutations.^13^ Moreover, *NF1* mutations are not just considered as lung cancer driver genes. In lung cancer models, resistance to *EGFR* targeted therapy was mediated by *NF1* expression, and blocking MEK restored the response.^14^

Deleterious *NF1* mutations have been previously reported in non‐small‐cell lung cancer (NSCLC), but the genomic landscape of this molecular subgroup remains poorly characterized in the lung cancer population; the clinical outcomes of *NF1* mutant NSCLC also remain unknown. In the present study, we retrospectively analyzed the clinicopathological characteristics, molecular profiles, and prognostic features of *NF1* mutations in *EGFR* mutant lung cancer patients.

## 2 TECHNOLOGY AND METHODS

### 2.1 Patients

From June 2016 to December 2020, 3230 samples from lung cancer patients from Guangdong Lung Cancer Institute (GLCI) were screened, analyzed, and sequenced for *NF1* mutations. Clinical data from these patients were retrospectively collected through a review of their electronic medical records. Factors included in the analysis were sex, age, date of diagnosis, smoking status at diagnosis, pathological type, and clinical stage at the time of diagnosis. The date of death was determined from regular follow‐up in the electronic medical records.

### 2.2 Study design

**FIGURE 1:**
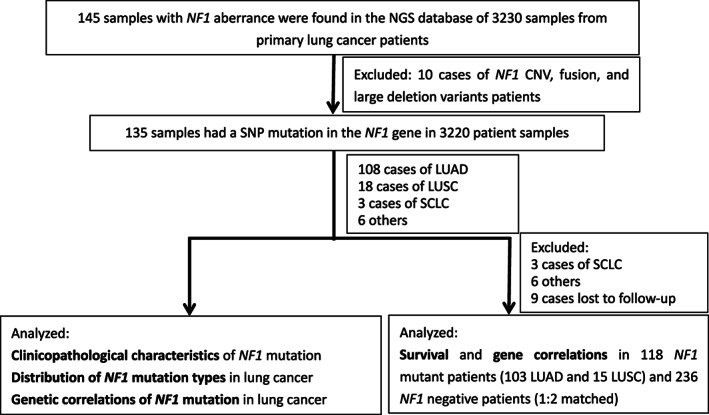
Flow chart of the study

This study involved 3230 samples from lung cancer patients (Figure 1). All specimens were subjected to *NF1* mutation analysis by next‐generation sequencing (NGS). For patients with multiple screenings, the results of initial tissue samples were prioritized, followed by blood, pleural effusion, and cerebrospinal fluid. The incidences of *NF1* mutations and the distributions of *NF1* mutation types were analyzed; the clinical characteristics and concurrent alterations of *NF1* mutations were evaluated in NSCLC patients. Additionally, 118 patients harboring *NF1* mutations and 236 *NF1*‐negative patients (1:2 matched according to sex, age, date of diagnosis, smoking status at diagnosis, pathological type, and clinical stage at the time of diagnosis) were analyzed for survival analysis and gene correlation analysis. The genes of interest in the study included *NF1*, *TP53*, *EGFR*, *RB1*, *KEAP1*, *ALK*, *RET*, *KRAS*, *BRAF*, *CDKN2A*, *PTEN*, *ROS1*, *PIK3CA*, *STK11*, *NRAS*, and *RASA1*.

### 2.3 Targeted sequencing

Tissue DNA was extracted using the QIAamp DNA FFPE Tissue Kit (Qiagen); cell‐free DNA in the plasma, pleural effusion, and cerebrospinal fluid was extracted using the QIAamp Circulating Nucleic Acid Kit (Qiagen). Purified DNA was hybridized with oligonucleotide baits; targeted capture was performed using a panel of 520 cancer‐related genes (OncoScreen Plus; Burning Rock Biotech., Ltd) or a panel of 425 cancer‐related genes (Nanjing Geneseeq Technology., Ltd.). The probes of the two company kits covered the *NF1* gene sequence. Samples were sequenced using the Illumina HiSeq 4000 platform or the NovaSeq 6000 platform (Illumina) with 2 × 150 base pair cycles at target sequencing depths of 1000× for tissue DNA samples and 10,000× for cell‐free DNA samples. Paired white blood cells were sequenced to filter out clonal hematopoiesis‐related mutations. The processing of sequencing data was performed using optimized bioinformatics pipelines to analyze various cancer‐related somatic mutations at the DNA level, including point mutations, insertions/deletions, copy number variations, and gene rearrangements. Protocols from previous publication were followed for both experimental procedures and sequence data analysis.^15^, ^16^ Table S1 showed the sequencing coverage and quality statistics of the next generation sequencing generated of *NF1* mutant patients in this study.

### 2.4 Statistical analysis

Analysis of associations between *NF1* gene mutations and clinical factors was performed using the chi‐squared test or Fisher's exact test using the Statistical Package for the Social Sciences Software (SPSS) version 25.0. Survival analysis was performed using graphPad prism Software version 9.0. Overall survival (OS) was measured from the date of pathological diagnosis of lung cancer to the date of death or last follow‐up, with a cut‐off date of August 31, 2021. Kaplan–Meier survival curves were generated to estimate OS in different genomic groups.

## 3 RESULTS

### 3.1 Clinicopathological characteristics of patients with *NF1* mutations

The clinical and pathological characteristics of Chinese lung cancer patients with *NF1* mutations and wild‐type *NF1* were compared (Table 1). Inactivating somatic *NF1* mutations were present in 4.0% (108/2696) of the LUAD cases and 6.5% (18/279) of the lung squamous cell carcinomas (LUSC) cases. The median of the *NF1* mutant and wild‐type patients were 64 and 59 years old, respectively. Patients with *NF1* mutations were more likely to be elderly (63.7% vs. 42.3%, *p* < 0.001). The proportion of men was higher in the *NF1* mutant patients than in patients without *NF1* mutations (72.6% vs. 57.1%, *p* < 0.001). 52.6% of *NF1* mutation patients were smokers, compared with 33.0% in the *NF1* wild‐type subgroup (*p* < 0.001). There were no significant differences in terms of pathological type or stage.

**TABLE 1:** Summary of demographic and clinicopathological characteristics of lung cancer patients with defined *NF1* mutations

Characteristics	Total	*NF1* status		
		Pos, no. (%)	Neg, no. (%)	*p*‐value
No. of patients	3220	135 (4.2%)	3085 (95.8%)	
Age (years)				
Median	59	64	59	<0.001
Range	17–92	35–87	17–92	
≤60	1828 (56.8%)	49 (36.3%)	1779 (57.7%)	
>60	1392 (43.2%)	86 (63.7%)	1306 (42.3%)	
Sex				
Male	1861 (57.8%)	98 (72.6%)	1763 (57.1%)	<0.001
Female	1359 (42.2%)	37 (27.4%)	1322 (42.9%)	
Smoking history				
Never‐smoker	2104 (65.3%)	62 (45.9%)	2042 (66.2%)	<0.001
Ever‐smoker	1088 (33.8%)	71 (52.6%)	1017 (33.0%)	
Unknown	28 (0.9%)	2 (1.5%)	26 (0.8%)	
Pathological types				
Adenocarcinoma	2696 (83.7%)	108 (80%)	2588 (83.9%)	0.053*
Squamous	279 (8.7%)	18 (13.3%)	261 (8.5%)	
SCLC	54 (1.7%)	3 (2.2%)	51 (1.7%)	
Other	135 (4.2%)	3 (2.2%)	132 (4.3%)	
Unknown	56 (1.7%)	3 (2.2%)	53 (1.7%)	
Stage				
I–IIIa	425 (13.2%)	20 (14.8%)	405 (13.1%)	0.563
IIIb–IV	2730 (84.8%)	112 (83.0%)	2618 (84.9%)	
Unknown	65 (2.0%)	3 (2.2%)	62 (2.0%)	

*Note*: Never‐smokers were patients who smoked less than 100 cigarettes in their lifetime.

Abbreviations: *NF1*, Neurofibromin; SCLC, small cell lung cancer.

* Comparison between adenocarcinoma and squamous; Statistical analysis excluded “unknown” subgroup.

### 3.2 Distribution of *NF1* mutation types

**FIGURE 2:**
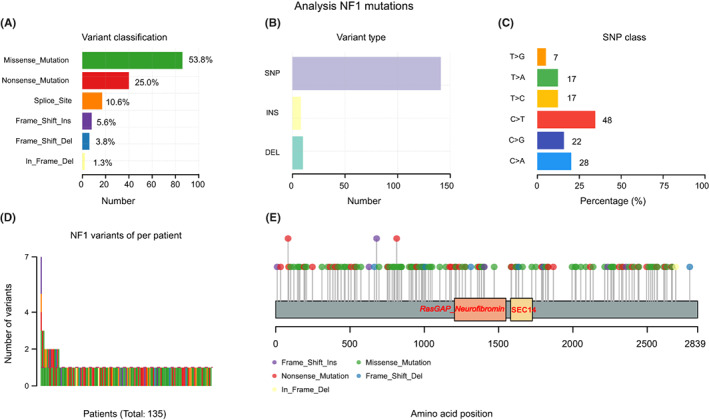
Distribution of all detected 160 *NF1* mutant types in our cohort of 135 lung cancer patients. (A–C) Variant classification, variant type, and single nucleotide polymorphism (SNP) class; (D) *NF1* variant number per patient; (E) The package “maftools” in R (version 4.0.2) was used to identify *NF1* variant sites in the amino acid sequence in our database.

All 160 *NF1* mutant sites were detected in our 135 *NF1* mutant lung cancer patients. Among these patients, missense mutations (53.8%), nonsense mutations (25.0%), and splice site mutations (10.6%) mainly occurred in the *NF1* gene (Figure 2A,B); the single base substitutions C > T and C > A were significantly prominent (Figure 2C). 11.9% (16/135) of patients had more than one *NF1* mutant sites; one patient harbored seven *NF1* mutations (Figure 2D). There were no hotspot mutations in the *NF1* genes; almost all of the 160 *NF1* mutant sites occurred only once and were spread throughout all exons of the *NF1* gene (Figure 2E).

### 3.3 Genetic co‐mutation of *NF1*

**FIGURE 3:**
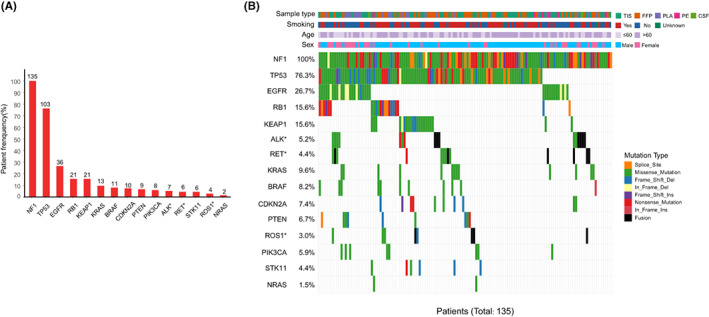
Genomic landscape of known oncogenes and suppressor genes in 135 *NF1* mutant patients. (A) Frequencies of selected known oncogenes and suppressor genes. (B) The waterfall function of GenVisR package in R (version: 4.0.3) was used to visualize the mosaic plot of the mutation landscape of selected genes. *Percentages of *ALK*, *ROS1*, and *RET* genes indicate fusion subtypes. TIS, tissue; FFPE, formalin‐fixed paraffin‐embedded tissue; PLA, plasma; PE, pleural effusion; CSF, cerebrospinal fluid.

*NF1* mutations frequently occurred with other oncogenic mutations in our study. The frequency of concurrent mutations and the mutation landscape of selected genes are presented in Figure 3. Among the 135 samples with *NF1* mutations, co‐mutations were found in the following genes of interest: *TP53*, 103 (76.3%); *EGFR*, 36 (26.7%); *RB1*, 21 (15.6%); *KEAP1*, 21 (15.6%); *KRAS*, 13 (9.6%); *BRAF*, 11 (8.2%); *CDKN2A*, 10 (7.4%); *PTEN*, 9 (6.7%); *PIK3CA*, 8 (5.9%); *ALK* fusion, 7 (5.2%); *RET* fusion, 6 (4.4%); *STK11*, 6 (4.4%); *ROS1* fusion, 4 (3.0%); and *NRAS*, 2 (1.5%). Figure S1 shows an overview of the genomic alterations in >8% of the co‐mutant genes in 135 *NF1* mutant samples: *TP53*, *EGFR*, *LRP1B*, *DPYD*, *NQO1*, *RB1*, *KEAP1*, *SPTA1*, *MTHFR*, *ALK*, *FAT1*, *EP300*, *XRCC1*, *RET*, *KRAS*, *ARID1A*, *SMARCA4*, *CTNNB1*, and *BRAF*.

Gene correlations in the *NF1* mutation population were compared between 118 patients with the *NF1* mutant NSCLC and 236 patients with *NF1* wild‐type NSCLC (Table 2). *NF1* mutant patients were more likely to have *TP53* (*p* = 0.003), *BRAF* (*p =* 0.001) and *RASA1* (*p* = 0.026) mutations; *NF1* mutations were mutually exclusive with *EGFR* mutations (*p* = 0.006). *KRAS* mutations were equally distributed in the *NF1* mutant and wild‐type groups (*p* = 0.615). Other co‐mutations also had similar frequencies between the two cohorts, including *ALK* (5.9% vs. 11.0%, *p* = 0.121), *RB1* (13.6% vs. 7.6%, *p* = 0.074), *PTEN* (6.8% vs. 3.8%, *p* = 0.219), *PIK3CA* (5.9% vs. 6.8%, *p* = 0.760), *CDKN2A* (8.5% vs. 6.8%, *p* = 0.564), *KEAP1* (15.3% vs. 8.5%, *p* = 0.052), *STK11* (3.4% vs. 6.8%, *p* = 0.193), *RET* (4.2% vs 2.1%, *p* = 0.311), *ROS1* (3.4% in both, *p* = 1.000), and *NRAS* (1.7% vs. 0%, *p* = 0.110).

**TABLE 2:** Gene correlations compared between 118 *NF1* mutant cases and 236 non‐*NF1* mutant cases in NSCLC patients

Gene	*NF1* wild‐type patients (*n* = 236)	*NF1* mutant patients (*n* = 118)	*p*‐value
*TP53*			
Mutation	138 (58.5%)	88 (65.2%)	0.003
Wild‐type	98 (41.5%)	30 (25.4%)	
*EGFR*			
Mutation	106 (44.9%)	35 (29.7%)	0.006
Wild‐type	130 (55.1%)	83 (70.3%)	
*KRAS*			
Mutation	22 (9.3%)	13 (11.0%)	0.615
Wild‐type	214 (90.7%)	105 (89.0%)	
*ALK*			
Fusion	26 (11.0%)	7 (5.9%)	0.121
Wild‐type	210 (89.0%)	111 (94.1%)	
*RB1*			
Mutation	18 (7.6%)	16 (13.6%)	0.074
Wild‐type	218 (92.4%)	102 (96.4%)	
*BRAF*			
Mutation	3 (1.3%)	11 (9.3%)	0.001*
Wild‐type	233 (98.7%)	107 (90.7%)	
*PTEN*			
Mutation	9 (3.8%)	8 (6.8%)	0.219
Wild‐type	227 (96.2%)	110 (93.2%)	
*PIK3CA*			
Mutation	16 (6.8%)	7 (5.9%)	0.760
Wild‐type	220 (93.2%)	111 (94.1%)	
*CDKN2A*			
Mutation	16 (6.8%)	10 (8.5%)	0.564
Wild‐type	220 (93.2%)	108 (91.5%)	
*KEAP1*			
Mutation	20 (8.5%)	18 (15.3%)	0.052
Wild‐type	216 (91.5%)	100 (84.7%)	
*STK11*			
Mutation	16 (6.8%)	4 (3.4%)	0.193
Wild‐type	220 (93.2%)	114 (96.6%)	
*RET*			
Fusion	5 (2.1%)	5 (4.2%)	0.311*
Wild‐type	231 (97.9%)	113 (95.8%)	
*ROS1*			
Fusion	8 (3.4%)	4 (3.4%)	1.000*
Wild‐type	228 (96.6%)	114 (96.6%)	
*RASA1*			
Mutation	1 (0.4%)	4 (3.5%)	0.026
Wild‐type	235 (99.6%)	114(96.6%)	
*NRAS*			
Mutation	0 (0.0%)	2 (1.7%)	0.110*
Wild‐type	236 (100%)	116 (98.3%)	

* *p*‐value calculated using Fisher's exact test.

### 3.4 Prognostic features of *NF1* mutant patients

**FIGURE 4:**
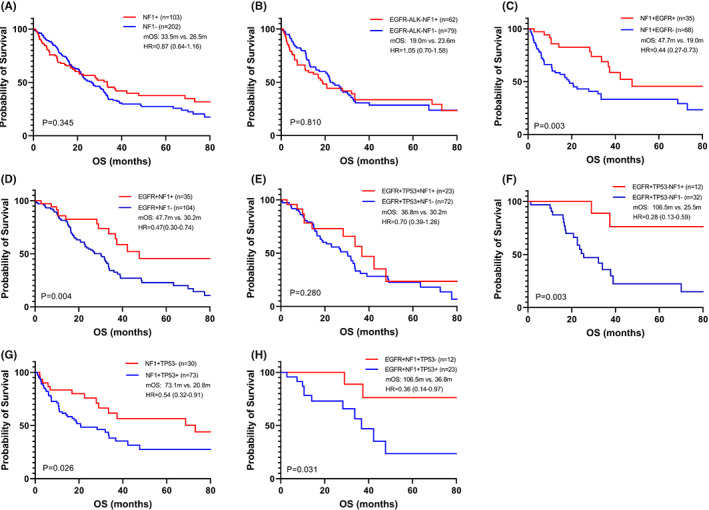
Overall survival in each subgroup of our patients. (A) Overall survival compared between *NF1*+ and *NF1*‐ cases in LUAD patients; (B) Overall survival compared between *NF1*+ and *NF1*‐ cases in LUAD patients without *EGFR* mutations and *ALK* fusion; (C) Overall survival compared between *NF1* + *EGFR*+ and *NF1* + *EGFR*‐ cases in LUAD patients; (D) Overall survival compared between *EGFR* + *NF1*+ and *EGFR* + *NF1*‐ cases in LUAD patients; (E) Overall survival compared between *NF1*+ and *NF1*‐ cases in *EGFR* + *TP53*+ LUAD patients; (F) Overall survival compared between *NF1*+ and *NF1*‐ cases in *EGFR* + *TP53*‐ LUAD patients. (G) Overall survival compared between *TP53*+ and *TP53*‐ cases in *NF1*+ LUAD patients. (H) Overall survival compared between *TP53*+ and *TP53*‐ cases in *EGFR* + *NF1*+ LUAD patients. mOS, median overall survival; HR, hazard ratio; +, mutation; −, wild‐type.

The median overall survival of 354 lung cancer patients enrolled in the study was 27.0 m (Figure S3A). Different *NF1* mutant types had no effect on OS (Figure S2A); there was no effect on OS between one and more than one *NF1* mutant sites (Figure S2B). *NF1* mutations had no effect on OS in all patients (Figure S3B); they also had no effect on median OS (mOS) in cases of LUAD (mOS: 33.5 m vs. 26.5 m, hazard ratio [HR] = 0.87, 95% CI: 0.64–1.16, *p* = 0.345; Figure 4A) and cases of LUSC (Figure S3C), respectively. Furthermore, *NF1* mutations had no effect on OS in LUAD cases who were driver gene‐negative (without *EGFR* mutations and *ALK* fusion) (mOS: 19.0 m vs. 23.6 m, HR = 1.05, 95% CI: 0.70–1.58, *p* = 0.810; Figure 4B). In LUAD cases, *NF1*/*EGFR* co‐mutant patients had significantly longer OS than *NF1* mutant/*EGFR* wild‐type patients (mOS: 47.7 m vs. 19.0 m, HR = 0.44, 95% CI: 0.27–0.73, *p* = 0.003). *EGFR*/*NF1* co‐mutant patients also had significantly longer OS than *EGFR* mutant/*NF1* wild‐type patients (mOS: 47.7 m vs. 30.2 m, HR = 0.47, 95%CI: 0.30–0.74, *p* = 0.004) (Figure 4C,D). *NF1* mutations had no effect on OS in LUAD patients with *EGFR*/*TP53* co‐mutations (mOS: 36.8 m vs. 30.2 m, HR = 0.70, 95% CI: 0.39–1.26, *p* = 0.280) (Figure 4E). However, *NF1* mutations had a significant effect on OS in LUAD patients with *EGFR* mutant/*TP53* wild‐type (mOS: 106.5 m vs. 25.5 m, HR = 0.28, 95% CI: 0.13–0.59, *p* = 0.003; Figure 4F). Table 3 showed the related clinical and genetic information in 12 cases of *NF1* mutant LUAD patients with *EGFR* mutant/*TP53* wild‐type. Since there were fewer LUSC patients with *NF1* mutation (n = 15), subgroup analysis cannot be performed in LUSC patients.

**TABLE 3:** The clinical and related genetic information in 12 cases of LUAD patient with *NF1* mutant/*EGFR* mutant/*TP53* wild‐type

No	Sex	Age	Smoking	Stage	OS (m)	Status	*KRAS*	*ALK*	*RET*	*NRAS*	*ROS1*	*BRAF*	*PIK3CA*	*RB1*	*CDKNA*	*KEAP1*	*STK11*	*PTEN*	Therapy
1	F	64	No	IA	199.2	death	−	−	−	−	−	−	−	−	−	−	−	−	Surg; Targ; Chem
2	M	64	Yes	IB	89.0	death	−	−	−	−	−	−	−	−	−	−	−	−	Surg; Targ
3	M	65	No	IIA	78.6	live	−	−	+	−	−	−	−	−	−	−	−	−	Surg; Targ; Chem
4	F	68	No	IIIB	27.0	live	−	−	−	−	−	−	−	−	−	+	−	−	Targ; Chem
5	M	71	No	IVA	106.5	death	−	−	−	−	−	−	−	+	−	−	−	−	Targ; Chem
6	F	65	No	IVA	40.5	live	−	−	−	−	−	−	+	−	−	−	−	−	Targ
7	M	59	No	IVA	29.0	death	−	−	−	−	−	−	−	−	−	−	−	−	Targ
8	F	68	No	IVA	25.9	live	−	−	−	−	−	−	−	−	−	−	−	−	Targ
9	F	59	No	IVA	22.4	live	−	−	−	−	−	−	−	−	−	−	−	−	Targ; Chem
10	F	63	No	IVB	107.5	live	−	−	−	−	−	−	−	−	−	−	−	−	Targ; Chem
11	M	39	Yes	IVB	37.4	death	+	−	−	−	−	−	−	−	−	−	−	−	Targ
12	M	67	No	IVB	37.0	live	−	−	−	−	−	−	−	−	−	−	−	−	Targ; Chem

Abbreviations: Chem, chemotherapy; F, female; LUAD, lung adenocarcinoma; M, male; Surg, surgery; Targ, targeted therapy for *EGFR* mutation.

*TP53* mutation had worsen prognosis in cases of *NF1* mutant (mOS: 73.1 m vs. 20.8 m, HR = 0.54, 95% CI: 0.32–0.91, *p* = 0.026) or *EGFR*/*NF1* co‐mutant (mOS: 106.5 m vs. 36.8 m, HR = 0.36, 95% CI: 0.14–0.97, *p* = 0.031) LUAD patients (Figure 4G,H). Genes with a co‐mutant rate of more than 8% in *NF1* mutant patients were enrolled for survival analysis (Figure S4). *NF1*/*CTNNB1* co‐mutant had better effect on OS in NSCLC patients (*p* = 0.027). *LRP1B*, *RB1*, *KEAP1*, *FAT1*, *KRAS*, *ARID1A*, and *SMARCA4* mutation had no effect in cases of *NF1* mutant NSCLC patients, respectively.

### 3.5 Prognostic features of *NF1* mutant patients in the TCGA database

*NF1* mutations had no effect on OS in LUAD patients in the TCGA database (mOS: 31.2 m vs. 46.7 m, HR = 1.62, 95% CI: 0.72–3.63, *p* = 0.156) (Figure S5A). *NF1* mutations also had no effect on OS in LUAD patients without *EGFR* mutations and *ALK* fusion (mOS: 31.2 m vs. 76.2 m, HR = 1.90, 95% CI: 0.80–4.53, *p* = 0.062; Figure S5B). Unfortunately, there was only one patient with *NF1*/*EGFR* co‐mutation, and there were no NF1 mutations in patients with EGFR mutant/TP53 wild‐type adenocarcinoma.

## 4 DISCUSSION

Genetic and molecular profiling of NSCLC has led to the discovery of actionable oncogenic driver alterations, which has revolutionized treatment for lung cancer. There are increasing efforts to investigate the oncogenic events and drug resistance mechanisms of targeted therapy behind the “driver‐related gene‐negative” cohort. In lung cancer, *NF1* gene mutations occur in approximately 7.0%–11.8% cases of LUAD^7^, ^8^, ^9^, ^13^, ^17^, ^18^ and 10.3%–12.0% cases of LUSC in Western population.^8^ In our data set, somatic *NF1* mutations were present in 4.0% (108/2696) cases of LUAD and 6.5% cases (18/279) of LUSC. The incidence of *NF1* mutations was significantly lower in Asia than in Western populations. This difference indicates that ethnicity may be an important determining factor, similar to findings regarding *EGFR* and *KRAS* mutations. Our data showed that the clinicopathological features of lung cancer patients with *NF1* mutations were clearly enriched in older (*p* < 0.001), male (*p* < 0.001), and smoking (*p* < 0.001) patients. Tlemsani et al. also reported that most patients with *NF1* alterations were men and smokers with LUAD.^19^ The occurrence of *NF1* mutations was not associated with the pathological stage (*p* = 0.563) in our study.

*NF1* mutations occurred with other oncogenic mutations in our study. As shown in Table 2, *NF1* mutant patients exhibited concurrent *TP53*, *BRAF*, or *RASA1* mutations; *NF1* mutations were mutually exclusive with *EGFR* mutations in 118 *NF1* mutant NSCLC patients and 236 *NF1* wild‐type NSCLC patients. Although *NF1* negatively regulates the RAS proto‐oncogene, no difference was observed in terms of *KRAS* mutations between the *NF1* mutant and wild‐type cohorts. Redig et al. reported that *NF1* mutations occur more frequently with *TP53* mutations and other oncogenic alterations in lung cancer patients.^18^ Mice that carry linked germline mutations in *NF1* and *TP53* develop malignant peripheral nerve sheath tumors.^20^, ^21^ In the present study, *TP53* mutations were obviously enriched in the *NF1* mutant lung cancer patients (103/135, 76.3%), with 71.3% (77/108) in LUAD cases and 100% (18/18) in LUSC cases. Our study showed *TP53* mutation had worsen prognosis in cases of *NF1* mutant or *EGFR*/*NF1* co‐mutant LUAD patients. Thus far, most studies^18^, ^19^, ^22^ concerning the genetic correlations of *NF1* mutations have been descriptive; they have not involved statistical analysis. The present study clearly demonstrated a relationship between *NF1* mutations and various oncogenes and suppressor genes of lung cancer.

*NF1* mutations were reported as potential biomarkers for poor prognosis in pancreatic ductal adenocarcinoma,^23^ colorectal cancer.^24^ In our study, 118 *NF1* mutant patients and 236 *NF1* wild‐type patients had enrolled for survival analysis. Our results showed that *NF1* mutations had no direct effect on OS in LUAD cases or LUSC cases, even in lung cancer patients without driver genes (*EGFR* mutations and *ALK* fusion). However, *EGFR*/*NF1* co‐mutations caused a significantly longer OS than a single mutation of either the *EGFR* or *NF1* gene in NSCLC patients. Thus, *NF1* mutations served as a good prognostic factor in patients with *EGFR* mutation, particularly in patients with *EGFR* mutant/*TP53* wild‐type patients. At present, limited data are available concerning *NF1* mutant patient prognosis. The prognostic features of *NF1*/*EGFR* co‐mutations have rarely been reported. Pan et al. reported that NF1 mutant patients had distinctive survival features; they may not benefit from EGFR tyrosine kinase inhibitor treatment.^22^ However, we considered that evidence to be marginally useful because there was only one patient. Furthermore, our survival analysis results were verified after comparison with the TCGA database. The OS of TCGA patients with *NF1* mutations (n = 25) indicated a trend of poor prognosis. Nevertheless, no conclusions could be drawn because of the statistically insignificant OS benefit; subgroup analysis cannot be performed because there are few samples in TCGA database. This year, Negrao et al. from the Anderson Cancer Center^25^ reported that *NF1* mutant/high tumor mutational burden (TMB) NSCLC patients had longer progression‐free survival than *NF1* wild‐type/high TMB NSCLC patients in a large‐scale study of PD‐L1 immune checkpoint inhibitor therapy. The *NF1* mutant NSCLC showed robust sensitivity to immune checkpoint inhibitor therapy, compared with *NF1* wild‐type NSCLC. Therefore, *NF1* mutations should not always be considered a poor biomarker in lung cancer, similar to mutations in the *KRAS* gene. The study by Negrao et al. and the present study may provide a new perspective concerning *NF1* mutations.

It's worth mentioning that 14 patients had *RASA1* mutations among 938 tested lung cancer patients (1.5%) in this study; *RASA1* gene was detected only by the panel of 520 cancer‐related genes. However, there were 5 cases of *RASA1* mutations among 59 *NF1* mutation patients (8.5%), which indicated that the occurrence of *RASA1* mutations is obviously related to the occurrence of *NF1* mutations in lung cancer patients. All 5 *RASA1*/*NF1* co‐mutation patients had *TP53* mutations; all patients lacked other known driver genes containing *EGFR*, *KRAS*, *ALK*, *ROS1*, or *RET*. Hayashi and colleagues^26^, ^27^ demonstrated that targeting downstream MAPK signaling with MEK inhibition in vitro was significantly more potent in NSCLC cells with *RASA1*/*NF1* co‐mutations than a single mutation of either *RASA1* gene or *NF1* gene. In our study, the OS in three adenocarcinoma patients with *RASA1*/*NF1/TP53* co‐mutations was 1 month (Stage IVA), 3 months (Stage IVB), and 31 months (Stage IIIB). One adenosquamous carcinoma patient (Stage IVA) has survived for 13 months of follow‐up, while one case was lost to follow‐up. Survival analysis could not be completed because of the small number of *RASA1*/*NF1* patients in our study. Luo J et al. reported the *NF1*, *TP53*, and *RB1* triple combined knockout zebrafish displayed severe developmental disruption.^28^ Here, we observed 14 cases of patients harboring *NF1*, *TP53*, and *RB1* co‐mutations; *RB1*/*NF1*/*TP53* co‐mutant patients showed no effect on OS (Figure S4).

There were several limitations in this study. First, this was a retrospective analysis. Second, this study only presents the results of survival analysis and fails to clarify the mechanism. At present, the mechanism of *NF1* mutations in lung cancer initiation and progression has not been explored. A preclinical study showed that *NF1* mutation‐induced RAS‐MAPK signaling activation could be effectively inhibited by MEK inhibitor therapy in NF1 patients.^7^, ^29^ The effectiveness of MEK inhibitors in NSCLC patients harboring *NF1* mutations has not yet been elucidated. An ongoing phase II trial examining the use of MEK inhibitor (trametinib) in patients with metastatic or locally advanced NSCLC harboring *NF1* mutations aims to answer this question (NCT03232892).^30^

## 5 CONCLUSION

This study was the largest comprehensive analysis for *NF1* gene in East Asia lung cancer patients. The frequency of *NF1* mutations in the Chinese population is significantly lower than that in western populations. *NF1* mutations were more common in older, male, and smoking lung cancer patients, and could define a specific population with a distinct clinical profile. *NF1* mutant patients enriched *TP53*, *BRAF*, and *RASA1* mutations, conversely, mutually exclusive with *EGFR* mutations. This study was more clearly showed the effect of *NF1* mutations on *EGFR* mutant lung cancer patients. *NF1* mutations served as a good prognostic factor in *EGFR* mutant/*TP53* wild‐type (not *EGFR*/*TP53* co‐mutation) lung cancer patients in this single‐center study. *NF1* mutations should not always be considered a poor biomarker in lung cancer. Additionally, *TP53* had worsen prognosis in cases of *NF1* mutant or *EGFR*/*NF1* co‐mutant LUAD patients.

## AUTHOR CONTRIBUTIONS

Conceptualization was performed by Hong‐xia Tian and Yi‐long Wu; methodology was performed by Hong‐xia Tian, Zhi‐hong Chen and Guang‐Ling Jie; formal analysis was performed by Hong‐xia Tian, Guang‐Ling Jie and Hong‐hong Yan; investigation was performed by Hong‐xia Tian, Si‐pei Wu and Zhen Wang; resources was performed by Yi‐long Wu; data curation was performed by Zhi‐hong Chen, Guang‐Ling Jie and Dan‐xia Lu; supervision was performed by Xu‐chao Zhang and Hong‐hong Yan; validation was performed by Yi‐long Wu and Xu‐chao Zhang; writing‐original draft preparation was performed by Hong‐xia Tian; writing‐review and editing was performed by Hong‐xia Tian, Shui‐lian Zhang and Guang‐Ling Jie.

## CONFLICT OF INTEREST

The authors declare no conflicts of interest.

## ETHICAL APPROVAL STATEMENT

The study protocol was approved by the Research Ethics Committee of Guangdong Provincial People's Hospital (No. KY‐Z‐2020‐260‐03). All patients provided written informed consent to participate.

## Supporting information


Data S1

## Data Availability

The data that support the findings of this study are openly available in TCGA at www.tcga.org. The data that supports the findings of this study are available from the corresponding author upon reasonable request.
