# Supplementary material for: Prognostic features and comprehensive genomic analysis of NF1 mutations in EGFR mutant lung cancer patients
Source: Cancer Med. 2022 Jun 14;12(1):396–406. doi: 10.1002/cam4.4925 (PMC9844590; doi:10.1002/cam4.4925)
Supplement: Supplementary file 1 — Data S1 [file CAM4-12-396-s001.docx]

SUPPORTING INFORMATION

**Prognostic features and comprehensive genomic analysis of *NF1* mutations**

**in *EGFR* mutant lung cancer patients**

**List of materials included in this document**

**1) Figure S1.** Waterfall plot of > 8% co-mutant genes in 135 *NF1* mutant samples.

**2) Figure S2.** Overall survival in *NF1* variant types of our patients.

**3) Figure S3.** Overall survival in each subgroup of our patients.

**4) Figure S4.** Overall survival compared in genes with a co-mutant rate of more than 8% in *NF1* mutant patients.

**5) Figure S5.** Overall survival in patients from the TCGA database.

**6) Table S1.** The sequencing coverage and quality statistics of the next generation sequencing generated of *NF1* mutant patients in this study.


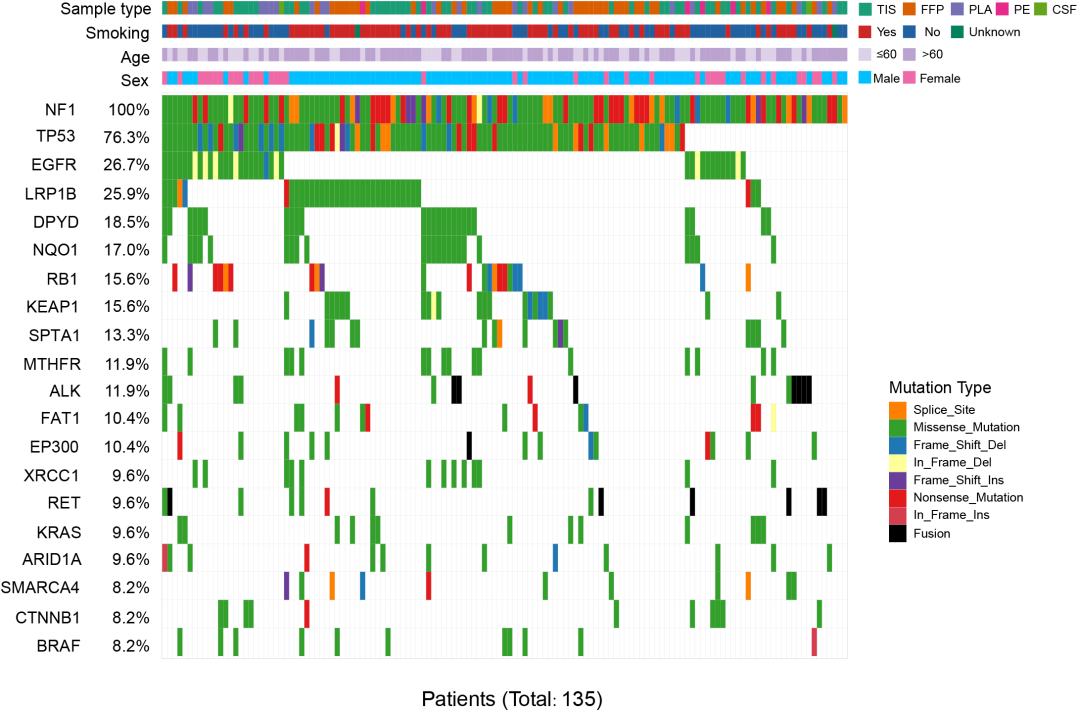


FIGURE S1 Waterfall plot of > 8% co-mutant genes in 135 *NF1* mutant samples

Note: (1) TIS: tissue; FFPE: formalin-fixed paraffin-embedded tissue; PLA: plasma; PE: pleural effusion; CSF: [cerebrospinal fluid](https://www.baidu.com/link?url=cvj7O491sMgDoAiiDG_VdXPijXE90KjxzGer5quN4a41lYTyG66p_T9M5YPDIXx2xbhOxXRzaA6efWOo_HFiNecmu4XM6ODMbGuQp8pbp-9jtvqaeZCbcxZDFqxh19II&wd=&eqid=bdf384a200012bbd0000000661800bfa). (2) Most genes were analyzed using all NGS kits except *DPYD*, *NQO1*, *MTHFR*, *XRCC1* and *SPTA1* genes. *DPYD*, *NQO1*, *MTHFR* and *XRCC1* were only detected by the panel of 425 cancer-related genes (73/135); *SPTA1* was only detected by the panel of 520 cancer-related genes (62/135).


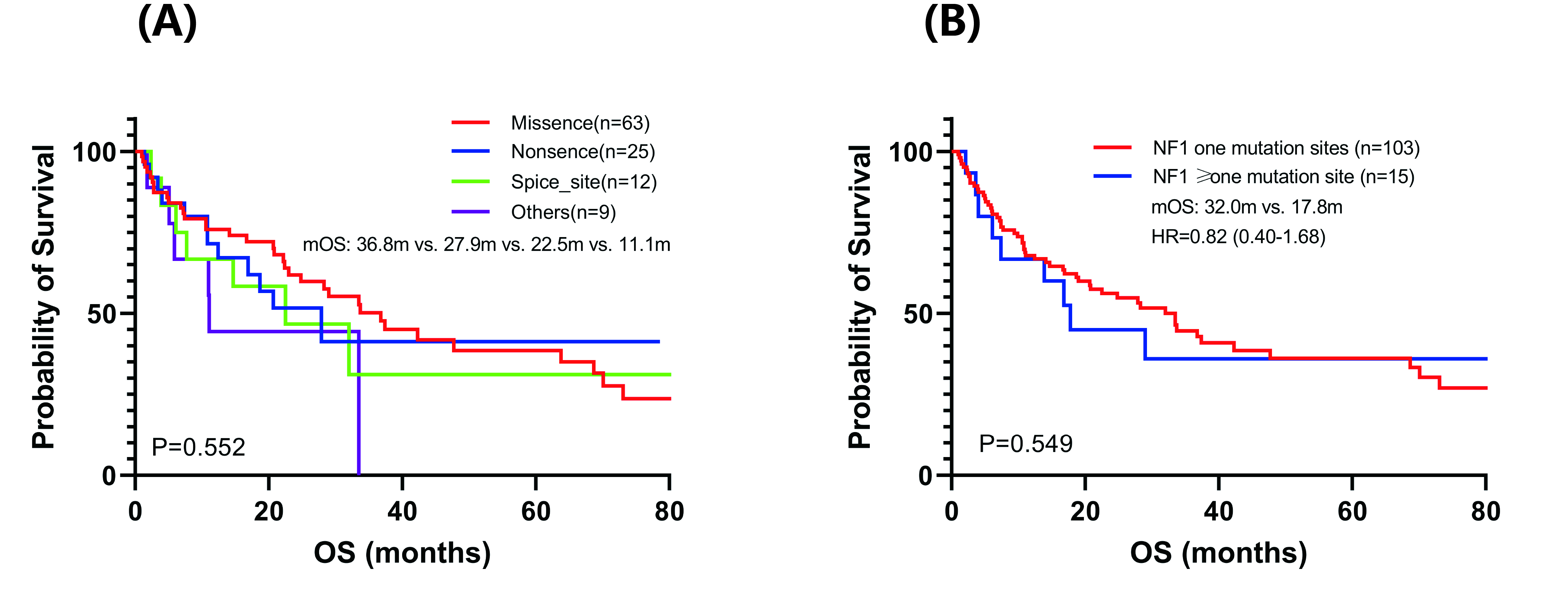


FIGURE S2 Overall survival in *NF1* variant types of our patients.

1. Overall survival compared among different *NF1* mutant types in NSCLC patients;
2. Overall survival compared between one and more than one *NF1* mutant sites in NSCLC patients.


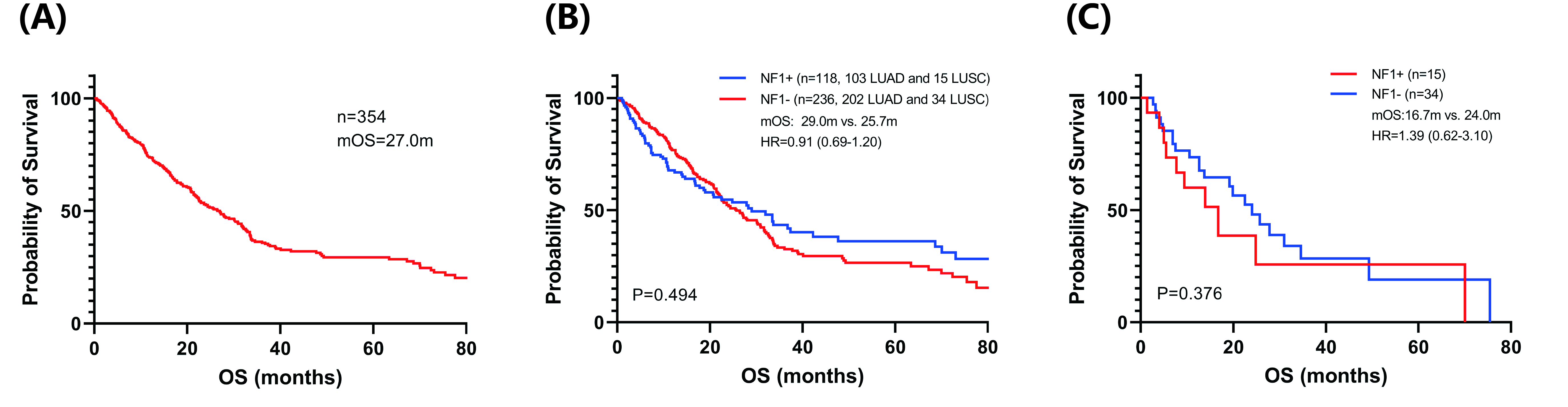


FIGURE S3 Overall survival in each subgroup of our patients.

(A)The median overall survival of 354 lung cancer patients enrolled in the study was 26.5 m (305 cases of LUAD and 49 cases of LUSC);

(B) Overall survival in all patients (n = 354, 305 cases of LUAD and 49 cases of LUSC);

(C) Overall survival compared between *NF1*+ and *NF1*- cases in LUSC patients;

Note: “+” represents mutation; “-” represents wild-type.

*LRP1B*

(A)


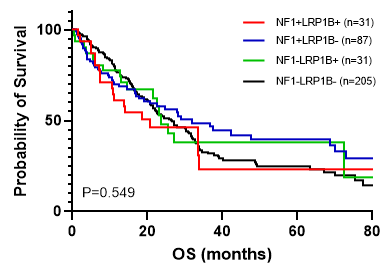


(A) Overall survival compared among different *LRP1B* and *NF1* cohorts of NSCLC patients.

*RB1*

(B)

(G)


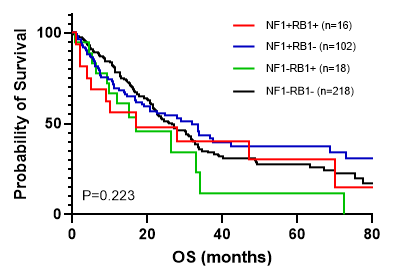


(B) Overall survival compared among different *RB1* and *NF1* cohorts of NSCLC patients.


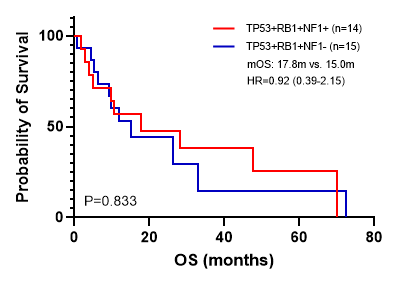


(C)

(C) Overall survival compared between *NF1*+ and *NF1*- cases in *TP53*+*RB1*+ NSCLC patients.


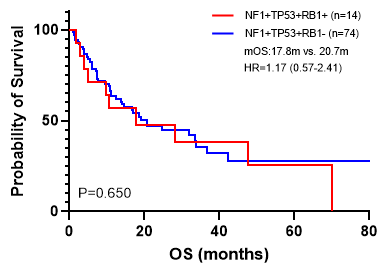


(D)

(D) Overall survival compared between *RB1*+ and *RB1*- cases in *NF1*+*TP53*+ NSCLC patients.

*KEAP1*

(E)


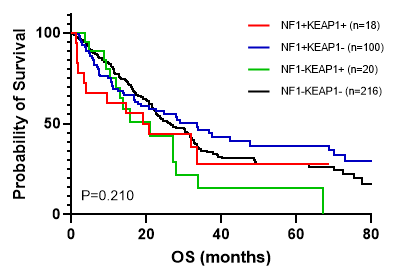


(E) Overall survival compared among different *KEAP1* and *NF1* cohorts of NSCLC patients.

*FAT1*

(F)


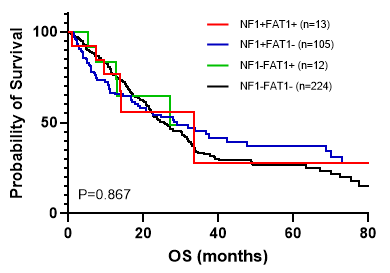


(F) Overall survival compared among different *FAT1* and *NF1* cohorts of NSCLC patients.

*KRAS*

(G)


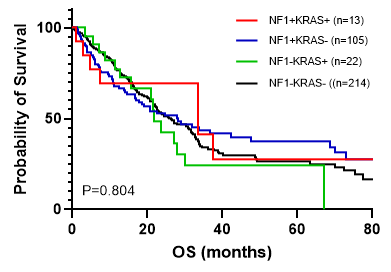


(G) Overall survival compared among different *KRAS* and *NF1* cohorts of NSCLC patients.

(H)


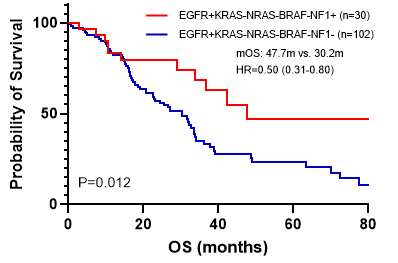


(H) Overall survival compared between *NF1*+ and *NF1*- cases in *EGFR*+*KRAS*-*NRAS*-*BRAF*- LUAD patients.

*ARID1A*

(I)


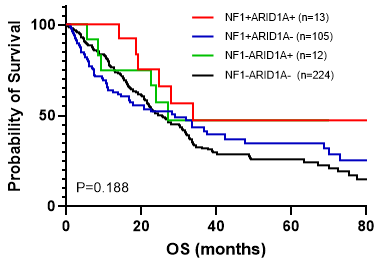


(I) Overall survival compared among different *ARID1A* and *NF1* cohorts of NSCLC patients.

*SMARCA4*

(J)


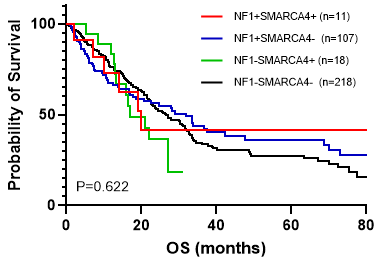


(J) Overall survival compared among different *SMARCA4* and *NF1* cohorts of NSCLC patients.

*CTNNB1*

(K)


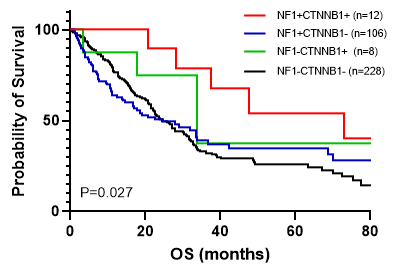


(K) Overall survival compared among different *CTNNB1* and *NF1* cohorts of NSCLC patients.

FIGURE S4 Overall survival compared in genes with a co-mutant rate of more than 8% in *NF1* mutant patients

Note: “+” represents mutation; “-” represents wild-type.


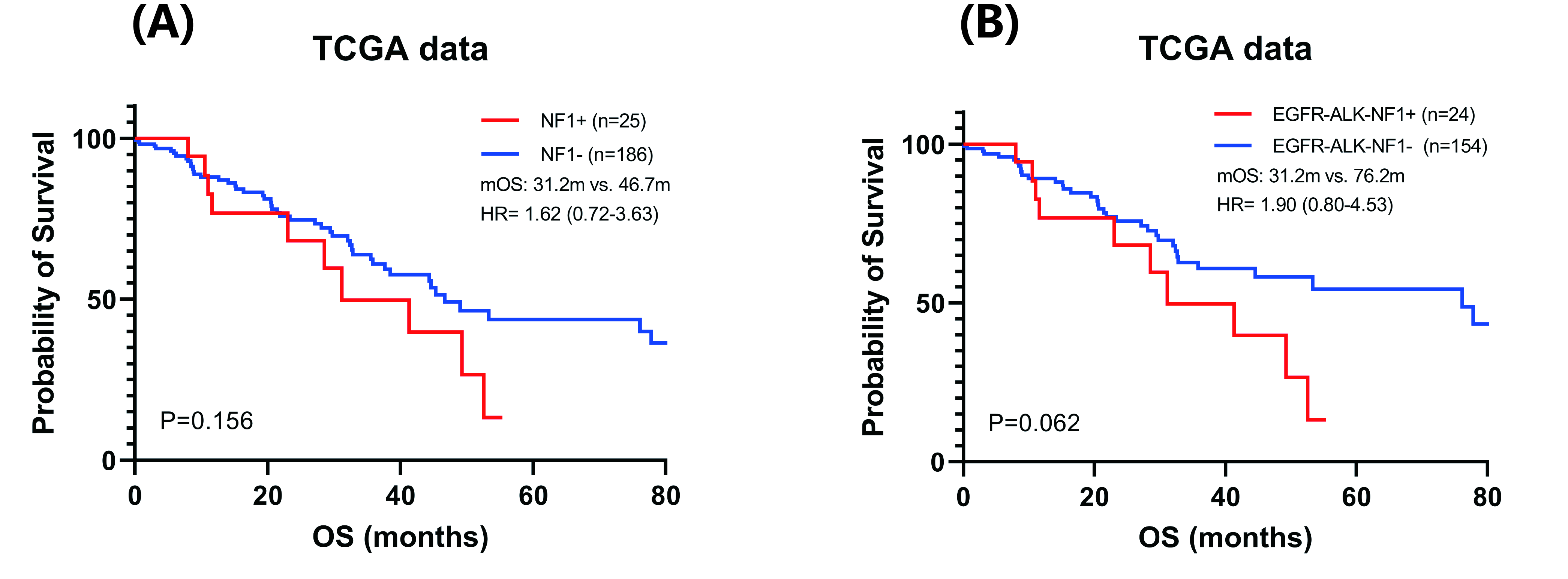


FIGURE S5 Overall survival in patients from the TCGA database.

1. Overall survival compared between *NF1*+ and *NF1*- cases in LUAD patients;
2. Overall survival compared between *NF1*+ and *NF1*- cases in LUAD patients without *EGFR* mutations and *ALK* fusion.

Note: “+” presents mutation; “-” presents wild-type.

TABLE S1  The sequencing coverage and quality statistics of the next generation sequencing generated of *NF1* mutant patients

| NO | Sample ID | Total number of sequenced reads | Total number of uniquely mapped non-duplicate reads^a^ | Total number of covered bases^b^ | Median coverage (and range) per base^b^ | Percentage of targeted bases with coverage ≥10^b,c,d^ |
| --- | --- | --- | --- | --- | --- | --- |
| 1 | RS1603608FFP | 31084369 | 24098115 | 415139046 | 590(1,5554) | 0.992999971 |
| 2 | RS1612108TIS | 29501676 | 14898346 | 305325093 | 700(1,3811) | 0.991999984 |
| 3 | RS1801236FFP | 19893454 | 4998111 | 82446211 | 195(1,34075) | 0.981999993 |
| 4 | RS1806914FLD | 50332194 | 37447153 | 254339755 | 1832(1,23059) | 0.992999971 |
| 5 | RS1810578TIS | 27909398 | 22104243 | 88462335 | 1187(1,3720) | 0.991999984 |
| 6 | RS1811814PLA | 49573484 | 20771290 | 207192073 | 7054(1,10846) | 1 |
| 7 | RS1818840PLA | 117440078 | 67880366 | 186009802 | 3877(1,33629) | 0.986000001 |
| 8 | RS1820737PLA | 130830834 | 65415417 | 200347446 | 3936(1,8933) | 0.986000001 |
| 9 | RS1821466TIS | 26616302 | 21186576 | 123739839 | 1120(1,4738) | 0.990999997 |
| 10 | RS1823260TIS | 26567262 | 20775599 | 194223931 | 999(1,6960) | 0.990999997 |
| 11 | RS1823833TIS | 28032100 | 25985756 | 66952680 | 1468(1,6033) | 0.995999992 |
| 12 | RS1826771FFP | 27354698 | 9217603 | 122313607 | 394(1,4347) | 0.985000014 |
| 13 | RS1828363FFP | 27830556 | 19787526 | 73004005 | 1031(1,5609) | 0.994000018 |
| 14 | RS1839920TIS | 30465136 | 17730710 | 121947192 | 932(1,2611) | 0.994000018 |
| 15 | RS1841240TIS | 29460580 | 25365559 | 73682281 | 1424(1,5314) | 0.995999992 |
| 16 | RS1844552PLA | 102100610 | 40636043 | 172145100 | 2168(1,16422) | 0.980000019 |
| 17 | RS19004112TIS | 28289360 | 21528202 | 67716263 | 1175(1,21557) | 0.995000005 |
| 18 | RS19005474FFP | 36027526 | 27489003 | 203928671 | 1362(1,6406) | 0.996999979 |
| 19 | RS19007658FFP | 32883346 | 25320176 | 148018596 | 1350(1,4384) | 0.995999992 |
| 20 | RS19009077FFP | 28157800 | 21259139 | 83492741 | 1147(1,5246) | 0.996999979 |
| 21 | RS19011882TIS | 23901672 | 19049632 | 63526465 | 1042(1,11272) | 0.995999992 |
| 22 | RS19012770PLA | 145801626 | 70859590 | 406325121 | 3889(1,51693) | 0.989000022 |
| 23 | RS19020763FFP | 27083728 | 21477396 | 446148742 | 851(1,24794) | 0.991999984 |
| 24 | RS19031173FFP | 28258240 | 20289416 | 69798304 | 1090(1,32185) | 0.995999992 |
| 25 | RS19032469TIS | 28282542 | 21042212 | 67963926 | 1099(1,27609) | 0.994000018 |
| 26 | RS19032501PLA | 193030790 | 71807456 | 166872968 | 4313(1,48245) | 0.992999971 |
| 27 | RS19035680FFP | 28609076 | 21628461 | 148587494 | 1088(1,3361) | 0.995000005 |
| 28 | RS19036259FFP | 29496718 | 21385121 | 502953343 | 805(1,7142) | 0.995000005 |
| 29 | RS19037651FFP | 28190736 | 19648944 | 135865799 | 1002(1,6366) | 0.995999992 |
| 30 | RS19039334FFP | 28504442 | 5168540 | 43874469 | 54(1,7036) | 0.981999993 |
| 31 | RS19041541FFP | 34123666 | 22043889 | 98218921 | 951(1,8340) | 0.998000026 |
| 32 | RS19044937PLA | 166494110 | 76087806 | 194104587 | 4567(1,12842) | 0.992999971 |
| 33 | RS19048609TIS | 22749278 | 19314137 | 109014936 | 974(1,8195) | 0.99000001 |
| 34 | RS19048875TIS | 28890516 | 23459098 | 337129322 | 1079(1,3260) | 0.995000005 |
| 35 | RS19059550TIS | 26195704 | 20694607 | 62194137 | 1113(1,4273) | 0.995000005 |
| 36 | RS19061903TIS | 37367668 | 30753591 | 262225933 | 1183(1,7385) | 0.995000005 |
| 37 | RS20001060PLA | 125260770 | 54738957 | 593807459 | 1830(1,8155) | 0.972000003 |
| 38 | RS20001477FFP | 31252814 | 25158515 | 252207986 | 942(1,4590) | 0.992999971 |
| 39 | RS20002899FFP | 35750786 | 26920343 | 275729020 | 1004(1,4853) | 0.995000005 |
| 40 | RS20005259TIS | 33591678 | 27645951 | 264814298 | 1146(1,5948) | 0.994000018 |
| 41 | RS20007109TIS | 24461776 | 21257284 | 291599747 | 848(1,5663) | 0.985000014 |
| 42 | RS20009889FFP | 34776320 | 28551358 | 250158022 | 1210(1,6979) | 0.994000018 |
| 43 | RS20010309FFP | 36198240 | 26135129 | 308428885 | 1050(1,5201) | 0.995999992 |
| 44 | RS20014642PLA | 48914714 | 19761545 | 211626921 | 6478(1,14924) | 0.995999992 |
| 45 | RS20016062PLA | 158410606 | 76829146 | 646775540 | 2960(1,10572) | 0.981000006 |
| 46 | RS20016785FFP | 32067416 | 26648022 | 257083474 | 1115(1,4888) | 0.992999971 |
| 47 | RS20017693TIS | 33101856 | 28103475 | 372130191 | 1066(1,9885) | 0.990999997 |
| 48 | RS20020332PLA | 177588840 | 88972007 | 617949226 | 3470(1,12595) | 0.981999993 |
| 49 | RS20026052PLA | 181744510 | 96869822 | 590760115 | 4202(1,11004) | 0.985000014 |
| 50 | RS20029183TIS | 31003406 | 25887843 | 351064963 | 1023(1,6574) | 0.994000018 |
| 51 | RS20029690TIS | 38083376 | 29666949 | 324206771 | 1183(1,4875) | 0.992999971 |
| 52 | RS20033121PLA | 173810786 | 100462636 | 743622943 | 4027(1,11391) | 0.981000006 |
| 53 | RS20038877FFP | 18176920 | 16104751 | 238489384 | 628(1,3563) | 0.984000027 |
| 54 | RS20039856FFP | 44173216 | 33439126 | 340186440 | 1362(1,7038) | 0.994000018 |
| 55 | RS20040031PLA | 162969896 | 105930429 | 873026106 | 4020(1,26899) | 0.976000011 |
| 56 | RS20040712PLA | 143401744 | 78153953 | 644248347 | 3130(1,10250) | 0.97299999 |
| 57 | RS20041426TIS | 37468676 | 30049878 | 324498726 | 1221(1,13954) | 0.994000018 |
| 58 | RS20044149TIS | 39601704 | 31087339 | 318299855 | 1263(1,5122) | 0.992999971 |
| 59 | RS20045785TIS | 32803544 | 27292549 | 360763009 | 1054(1,4417) | 0.985000014 |
| 60 | RS20047474FFP | 22129526 | 19100791 | 284857224 | 726(1,23602) | 0.987999976 |
| 61 | RS20051842FFP | 33714674 | 16638394 | 327397366 | 520(1,2537) | 0.995000005 |
| 62 | RS20056569FFP | 22466906 | 19164270 | 244207037 | 753(1,4590) | 0.989000022 |
| 63 | TA196N0202 | 9354546 | 5783030 | 921546748 | 603 | 99.88 |
| 64 | PA195W0257 | 27065143 | 13097247 | 2344764261 | 1629 | 99.89 |
| 65 | FB195T0135 | 13361689 | 7943283 | 1281183658 | 825 | 99.81 |
| 66 | FB19330080 | 13724410 | 1588388 | 249719652 | 178 | 99.6 |
| 67 | PA192T0044 | 5633508 | 2335214 | 437230099 | 4153 | 98.78 |
| 68 | F181230174401 | 6212488 | 4347496 | 635945492 | 462 | 99.84 |
| 69 | F181211168119 | 12973599 | 7617540 | 1181867062 | 878 | 99.89 |
| 70 | P181129164542 | 31109343 | 14939188 | 2793063109 | 2217 | 99.98 |
| 71 | FB195U0361 | 9119602 | 5582180 | 799987163 | 521 | 99.74 |
| 72 | F181026156035 | 1214966 | 806113 | 107583403 | 957 | 99.34 |
| 73 | P181011151960 | 27795550 | 13916105 | 2634115428 | 2049 | 99.97 |
| 74 | T180908145672 | 8430831 | 6238918 | 1029677624 | 758 | 99.86 |
| 75 | F180902144365 | 6465658 | 3966610 | 670729807 | 463 | 99.92 |
| 76 | C180705130901 | 1424974 | 989160 | 151655530 | 1216 | 99.87 |
| 77 | P180627129093 | 17823741 | 4314820 | 753763332 | 608 | 99.88 |
| 78 | T180624128603 | 1150334 | 832145 | 111456485 | 980 | 99.34 |
| 79 | T180510118302 | 8893440 | 6537215 | 1019303983 | 764 | 99.84 |
| 80 | C180421114330 | 7923596 | 6008325 | 1045533357 | 860 | 99.81 |
| 81 | F180410111640 | 7379964 | 5524629 | 912426541 | 723 | 99.77 |
| 82 | F18020399933 | 1227720 | 847742 | 118445422 | 1061 | 98.69 |
| 83 | T17071065971 | 1258857 | 893184 | 138538341 | 1159 | 100 |
| 84 | FB19AS0303 | 10939083 | 5146572 | 625890105 | 400 | 99.44 |
| 85 | FB19AN0074 | 9971063 | 6174408 | 976583223 | 617 | 99.81 |
| 86 | TA190V0006 | 11387661 | 7788151 | 1310324054 | 849 | 99.88 |
| 87 | TA190Q0211 | 7563283 | 4165039 | 663448187 | 342 | 99.96 |
| 88 | TA190N0153 | 9219377 | 5848714 | 943818545 | 619 | 99.79 |
| 89 | TA190K0094 | 1391386 | 780994 | 112904901 | 948 | 99.35 |
| 90 | TA190K0109 | 10102379 | 5264820 | 810290061 | 511 | 99.89 |
| 91 | TA190C0112 | 11869763 | 6749869 | 914371254 | 586 | 99.97 |
| 92 | CE190F0382 | 21401596 | 5732102 | 896206003 | 611 | 99.87 |
| 93 | TB190D0147 | 6129966 | 3807771 | 577444562 | 366 | 99.68 |
| 94 | FB199Q0400 | 10217658 | 4848600 | 820563731 | 498 | 99.96 |
| 95 | FB199J0394 | 20201268 | 10450452 | 1767392769 | 1130 | 99.85 |
| 96 | TA199A0144 | 12139482 | 5622434 | 713778941 | 459 | 99.83 |
| 97 | FB198D0209 | 13935038 | 7836295 | 1163482180 | 715 | 99.87 |
| 98 | PA19890297 | 36538861 | 16194927 | 3135909931 | 2180 | 99.91 |
| 99 | CA19830008 | 29143314 | 14849123 | 2783726181 | 1930 | 99.88 |
| 100 | TA197T0074 | 9269723 | 5594157 | 838904352 | 536 | 99.88 |
| 101 | TA197Q0114 | 9085862 | 5612940 | 828952190 | 538 | 99.87 |
| 102 | TA197A0171 | 13075356 | 7041866 | 1122694394 | 694 | 99.91 |
| 103 | TA198Q0055 | 6130082 | 4139474 | 651216236 | 430 | 99.86 |
| 104 | FB199U0142 | 15608937 | 8363159 | 1207865020 | 796 | 99.71 |
| 105 | TF180718133701 | 887076 | 424087 | 59006439 | 436 | 99.82 |
| 106 | FB205N0297 | 4001732 | 2106371 | 306953296 | 205 | 99.74 |
| 107 | TA205K0063 | 10723080 | 5936117 | 1035419348 | 687 | 99.92 |
| 108 | FB203P0164 | 3649012 | 1595050 | 181886341 | 104 | 96.72 |
| 109 | TA19BR0127 | 8731063 | 6207410 | 1023698887 | 681 | 99.84 |
| 110 | EA205B0133 | 10779116 | 1587483 | 224632408 | 147 | 99.88 |
| 111 | FB20AP0256 | 7061490 | 4459427 | 854088339 | 551 | 99.89 |
| 112 | TB20AE0164 | 30433546 | 18002720 | 3151415427 | 2028 | 99.98 |
| 113 | FB20A50566 | 13679313 | 9669964 | 1830832317 | 1159 | 99.9 |
| 114 | TB200U0205 | 11108531 | 7560772 | 1326341042 | 852 | 99.92 |
| 115 | TB209U0448 | 2770518 | 1900045 | 306451129 | 2740 | 100 |
| 116 | TA209C0133 | 6942364 | 5001951 | 867021765 | 593 | 99.85 |
| 117 | CE208U0344 | 47046810 | 8424209 | 1176167167 | 803 | 99.92 |
| 118 | TB20890060 | 12189200 | 8314002 | 1379463077 | 875 | 99.81 |
| 119 | TB208D0088 | 25973972 | 17023240 | 3006195712 | 1982 | 99.84 |
| 120 | TB20820131 | 22732746 | 16048886 | 2932920915 | 1538 | 99.77 |
| 121 | CA20720296 | 19087862 | 6103905 | 1021044073 | 9476 | 99.35 |
| 122 | TB206L0222 | 18722813 | 12785937 | 2343931261 | 1530 | 99.92 |
| 123 | TB206A0159 | 1411751 | 924413 | 149261681 | 1102 | 100 |
| 124 | TB20670132 | 10771577 | 6396224 | 1060058619 | 709 | 100 |
| 125 | TA205D0072 | 10222773 | 6265511 | 937768332 | 601 | 99.78 |
| 126 | TA190V0136 | 11864505 | 7682568 | 1260680160 | 765 | 99.95 |
| 127 | PA190N0293 | 14343455 | 4642948 | 753013386 | 6836 | 99.44 |
| 128 | TA197C0246 | 11322105 | 6335621 | 1005329039 | 634 | 99.89 |
| 129 | PA206A0111 | 22761717 | 4514962 | 746203850 | 6936 | 98.78 |
| 130 | PA206H0071 | 40539138 | 17087978 | 3157816377 | 2198 | 99.95 |
| 131 | FB194R0395 | 8018384 | 4596813 | 698652699 | 508 | 99.92 |
| 132 | TA188N0150 | 8069004 | 5680681 | 892113849 | 676 | 99.83 |
| 133 | CA193K0145 | 8147366 | 4712575 | 807532634 | 584 | 99.46 |
| 134 | CA201A0054 | 17747574 | 4408383 | 696231300 | 6319 | 99.35 |
| 135 | FB175H0017 | 8030155 | 2374064 | 275173512 | 293 | 99.35 |

^a^ Specify in table description or legend which reference genome was used (e.g., GRCh38).

^b^ After removing unmapped, non-uniquely mapped and duplicate reads.

^c^ Define "argeted bases" in table description or legend (e.g., whole genome, whole exome).

^d^ A higher minimum coverage threshold is permitted.
